# Knowledge on, Attitude Toward, and Practice of Contraceptive Methods Among Females of Reproductive Age in Al-Qunfudah Governorate, Saudi Arabia

**DOI:** 10.7759/cureus.36606

**Published:** 2023-03-23

**Authors:** Safa H Alkalash, Shroog M Alessi, Amal A Alrizqi, Amal A Alamri, Amnah Al Kenani, Hatim A Alrizqi, Rahaf Alqozi

**Affiliations:** 1 Community Medicine and Healthcare, Umm Al-Qura University, Al-Qunfudah, SAU; 2 Family Medicine, Menoufia University, Shebin Alkom, EGY; 3 Medicine and Surgery, Umm Al-Qura University, Al-Qunfudah, SAU

**Keywords:** saudi arabia, practice, attitude, knowledge, contraceptive

## Abstract

Background

One of the main goals of Vision 2030 in Saudi Arabia is to increase the participation rate of Saudi females in the workforce. This adjustment may have a big impact on how they use contraception and increase the tendency to space out their children's births appropriately to help them balance their home and work lives. The purpose of this study was to assess the knowledge on, attitude toward, and practice of contraceptive methods among females of reproductive age (15-49 years) in Al-Qunfudah governorate, Saudi Arabia.

Methods

A cross-sectional study was carried out among a convenient sample of 400 females of reproductive age in Al-Qunfudah governorate, Saudi Arabia. The necessary data were obtained over a period of two months (from November to December 2022) by using a self-administered online survey that was applied on different electronic platforms. Both knowledge and attitude scores were divided into two categories using the median as a cutoff point (e.g., good knowledge and poor knowledge, as well as positive and negative attitude). Many sociodemographic variables, such as age, residence, and education, were independent variables. Logistic regression analysis was done to determine the magnitude of associations between independent and dependent variables, and the odds ratios (OR) were presented with 95% confidence intervals (CI) at a significance level of P = 0.05.

Results

Good knowledge of the different contraceptive methods was observed among 69.8% of the females, where contraceptive pills and the intrauterine device (IUD) were the most well-known contraceptive methods to them (85.25% and 57.75%, respectively). Family and friends were their main sources of information (38.75%). Almost 85% of the participants showed a positive attitude toward contraceptive use. Contraceptive pills (32.39%) and IUDs (29.95%) were the most commonly used contraceptive methods. The determinants of good knowledge of contraception were being younger in age (P = 0.01, OR = 0.14, 95% CI = 0.03-0.65) and living in an urban area (P = 0.01, OR = 0.24, 95% CI = 0.09-0.68). Females holding middle or high school educational degrees (P = 0.02, OR = 0.17, 95% CI = 0.04-0.75 and P = 0.03, OR = 0.23, 95% CI = 0.06-0.88, respectively) and having a low monthly income (P = 0.04, OR = 0.44, 95% CI = 0.20-0.96) were likely to have positive attitudes toward contraceptive methods.

Conclusion

This study concludes that females of reproductive age had satisfactory knowledge and a positive attitude toward various contraceptives; however, there is a big gap in their knowledge regarding two important contraceptive procedures (emergency and permanent contraceptives). Oral contraceptive (OC) pills and IUDs were the most utilized methods of contraception among them. Sustained efforts are needed to raise females' awareness about contraception methods, especially emergency contraceptives and permanent ones. This study was done on a convenient sample of females in reproductive age that may limit the generalization of data; using an online survey has its constraints, such as the ignorance of the illiterate females and those who did not possess internet connections in addition to recall bias; therefore, we recommend further research on this topic through an interactive interview among a random sample of females to overcome such pitfalls.

## Introduction

According to the World Health Organization (WHO), one of the factors affecting females' health and empowerment in society is the frequency of contraceptive use. Contraceptives are methods to control pregnancies and space out births [1]. Birth control, also known as contraception, has reduced maternal mortality by 40% in the last 20 years simply by reducing the number of unintended pregnancies [2]. Increased contraceptive use has reduced the maternal mortality ratio (the risk of maternal death per 100,000 live births) by about 26% in less than a decade by preventing high-risk pregnancies, particularly in females of high parity, and those that would have resulted in unsafe abortion [2]. In order to reduce the risk of adverse maternal, prenatal, and infant outcomes, the WHO recommends that the interval between a live birth and the next pregnancy be 24 months [3]. The main purpose of contraceptive use is to help avoid unexpected pregnancies and facilitate family planning through the use of efficient and secure techniques [3].

In comparison to wealthy nations, Saudi Arabians have a high birth rate and a high total fertility rate; nevertheless, both rates have significantly decreased during the recent few years [4]. In general, there are many different ways to achieve contraception, including mechanical, chemical, hormonal, surgical, and natural methods. Mechanical contraceptives, such as the intrauterine device (IUD), which is inserted and remains in the uterus, prevent conception through several modes of action. IUDs may be medicated or non-medicated; examples include the inert Lippes Loop, Copper-T (medicated with copper), and Progestasert (medicated with progesterone) [5]. Hormonal contraceptives are available in many forms, such as pills, injections, patches, vaginal rings, and subdermal implants. The two types of oral contraceptives (OCs) that are widely used are combination OCs, consisting of estrogen and progestin, and the progestin-only pill (often called the minipill) [5]. Chemical contraceptives, such as spermicidal cream or gel, are available, while barrier contraceptives involve male and female condoms, which have an additional benefit. Besides their role as contraceptive methods, they also protect partners from sexually transmitted infections (STIs). In addition to natural methods of contraception, which require the avoidance of sexual relations during the fertile period [6], all previous forms of contraception were used as temporary contraceptives, while other forms such as a female's tubal ligation and a male's vasectomy act as permanent contraceptive methods [2].

Health advantages have been shown to exceed the dangers of using hormonal methods of contraception, despite the side effects associated with their use. These medications also provide a variety of therapeutic advantages, including the ability to treat polycystic ovarian syndrome, lower the risk of certain gynecological cancers, control menstruation, and lower the prevalence of sexually transmitted illnesses [5].

Understanding among females of their sexual lives, the important role of contraception in their family lives, and access to safe and effective methods are essential for better health [7]. Improving the knowledge and practice of both partners is necessary for achieving a consistent marital life [8]. Knowledge, attitude, and practice studies were begun in Asian nations in 1950, followed by the United States in 1955. Sociocultural factors and age, parity, education status, family attitude, and acceptability of contraception are among the factors that affect the utilization rate of different contraceptive methods [9]. A few studies were done in different areas of Saudi Arabia, such as Al-Madinah Al-Munawarah and Taif, concerning pregnancy spacing among Saudi females and detected a high level of knowledge and practice of contraceptives among their study subjects [10,11]. However, studies done in other Saudi areas such as Al-Qassim and Jeddah showed a low level of knowledge about this topic among females attending healthcare settings [12,13]. Despite the availability of various contraceptive methods, only 30.4% of Saudi females utilize any type of contraceptive method [14]. This study looked into females' tendencies toward using contraception and their knowledge of various contraceptive options in Al-Qunfudah governorate due to the special characteristics of this remote area being away from any central cities with limited healthcare settings and health education campaigns in comparison to other areas of Saudi Arabia.

## Materials and methods

Design of the study

A six-month cross-sectional study was conducted among a convenient sample of 400 females of reproductive age in Al-Qunfudah governorate, Saudi Arabia, from September 2022 to February 2023 (representing the whole study duration, starting from getting ethical approval to publication). The study targeted married females with reproductive ages of 15-49 years.

Study location

Al-Qunfudah governorate was selected purposively, which is a province of Makkah, and is the study location. It manifests itself in the Tihamah plain on Saudi Arabia's Red Sea coast. Its population has significant difficulties as a result of its remote location from any significant urban centers. It has numerous primary care facilities scattered throughout its cities and villages, as well as around three general hospitals.

Study sample

Based on the total number of females in the Al-Qunfudah governorate (125,206) and the frequency of contraception utilization from a previous Saudi study (53.5%) [15], the sample size was calculated using Epi Info™ (CDC, Atlanta, GA) [16], with a 95% confidence interval (CI) and a margin of error of 5%. The calculated sample size was 381.

Tool and procedure for data collection

A self-administrated survey was used to collect the required data. The questionnaire was developed by the study's researchers following a thorough literature review [11-13,15] and the research group's discussion to select the proper survey items. After constructing the survey, it was pretested in a pilot study to see whether it would be understandable by females of various educational backgrounds and levels, as well as to establish response rates. The survey link was disseminated on Al-Qunfudah Snapchat for a few days and then deliberately locked until we had finished the analysis of the preliminary data we had obtained, which in this pilot involved the first 40 submitted answers (representing 10% of the estimated sample size). When we evaluated the reliability of the survey, Cronbach's alpha coefficient was 0.86. The data from the pilot study were not included in the main study results.

There were 40 survey questions in total, broken down into four groups. Ten questions about the participants' sociodemographic and obstetric details were included in the first segment (age, residence, nationality, education, occupation, birth spacing, average monthly family income, duration of marriage, number of pregnancies, and living children). Eight questions were included in the second portion to assess their understanding of the numerous forms of contraceptives, the idea of family planning, the various side effects of contraceptive techniques, and the sources from which they had learned about those methods. The third component of the poll consisted of 15 questions to gauge respondents' attitudes on the advantages of contraceptives for both individuals and communities, as well as the role of the spouse in family planning technique selection. The fourth portion had seven items that discussed the various contraceptive techniques they have ever used.

With the distribution of the structured survey on various electronic platforms such as WhatsApp, Twitter, and Snapchat for Al-Qunfudah, the necessary study data were collected over a two-month period, from November to December 2022. To confirm that all data were obtained from Al-Qunfudah females, the survey asked each participant about their residence, and those from outside were eliminated and not included in the study's final conclusions. We received 522 responses, and after filtering them, we found 35 incomplete responses and 87 responses that came from outside the Al-Qunfudah governorate. These two were disregarded from the data analysis. Ultimately, 400 questionnaires were filled out in total.

The knowledge of different contraceptive methods was created using eight questions about different contraceptives. Each question's response was encoded as "1" for "correct" and "0" for "incorrect" answers. The participants' knowledge and attitude scores toward contraceptive methods were estimated using the median as a cutoff point. We categorized knowledge level into "good" (greater than or equal to the median score) and "poor" (less than the median score) and the same for positive and negative attitudes [17]. As regards knowledge score, a median score of less than 15 was considered poor knowledge, and a score of 15 or more was considered good knowledge. For the attitude score, a median attitude score of less than 13 was considered negative, while one of 13 or more was positive. Females were asked whether they had ever used any type of contraceptive method before this survey, and their responses were recorded as "yes" or "no" in addition to other questions about the methods used to assess their utilization of these methods.

The Medical Research and Ethical Committee of the College of Medicine, Umm Al-Qura University, Makkah, Saudi Arabia, issued the study the HAPO-02-K-012-2022-11-1291 ethical permission number before it was carried out. All study participants provided their informed consent in response to an introduction question at the start of the survey. Data were collected anonymously, and all information collected was kept confidential in secure files with a password.

Data analysis

Statistical Package for Social Sciences (SPSS) version 25.0 (IBM SPSS Statistics, Armonk, NY) was used for all statistical calculations. All data were entered, coded, and checked for consistency. Frequencies, percentages, medians, means, and standard deviations (SD) were used to show descriptive data. A binary logistic regression was done to identify the association between the independent variables (age, residence, nationality, education, occupation, birth spacing, average monthly family income, duration of marriage, number of pregnancies, and living children) and the dependent ones (knowledge and attitude scores). The odds ratio (OR) and 95% confidence interval (CI) were reported. P = 0.05 is regarded as statistically significant.

## Results

In the study, 400 females took part. Their ages were divided into four categories, with the age group 31-40 years having the edge with a percentage of 39.75%. Saudi participants made up 86.75% of the sample, 47% had bachelor's degrees, 50% were housewives, 33.5% were employees, and 48.25% had monthly incomes between 5,000 and 10,000 Saudi Arabian Riyal (SAR). In terms of maternal statistics, 63.5% had been married for more than one year up to 10 years, and 38% had a birth spacing of four to five years (Table 1).

**Table 1:** Demographics and maternity information of the participants (N = 400) All values are presented as numbers (N) and percentages (%) SAR: Saudi Arabian Riyal

Statement		N	%
Age group (years)	18-25	53	13.25
	26-30	97	24.25
	31-40	159	39.75
	41-49	91	22.75
Nationality	Saudi	347	86.75
	Non-Saudi	53	13.25
Residency	Ahad Bani Zayd	36	9.00
	Alardyat	55	13.75
	Al-Qunfudah City	157	39.25
	Al Quoz	83	20.75
	Haly	45	11.25
	Sabt Aljarah	24	6.00
Education	Reads and writes	13	3.25
	Elementary school	15	3.75
	Middle school	50	12.50
	High school	119	29.75
	University level	188	47.00
	Above university level	15	3.75
Employment status	Housewife	200	50.00
	Employee	134	33.50
	Student	66	16.50
Monthly income in SAR	<5,000	63	15.75
	5,000-10,000	193	48.25
	>10,000	144	36.00
The duration of marriage in years	≤1	57	14.25
	>1-10	254	63.50
	>10	89	22.25
Birth spacing in years	1	65	16.25
	2-3	141	35.25
	4-5	152	38.00
	>5	42	10.50
Number of pregnancies	None	29	7.25
	1-4	307	76.75
	>4	64	16.00
Number of live children	None	38	9.50
	1-4	272	68.00
	>4	90	22.50

Contraceptive pills were cited as the most well-known method of birth control by 85.25% of females, followed by IUDs with 57.75% and male condoms in third place with 34%. The most effective method of contraception was unknown to all participants. Only one-third of the survey participants knew that barrier contraceptives protect against the spread of STIs. Half of the study participants (48.75%) could not identify the permanent methods of birth control. The most common adverse reaction of contraception was mood swings or depression (72%), which was followed by vaginal infection/urinary tract infection (UTI) (58.75%) and menorrhagia (53.5%), acne vulgaris (52.75%), weight gain (52.25%), and unwanted pregnancy (51%). Amenorrhea and ectopic pregnancy were the least common adverse effects (31.25% and 30.75%, respectively). The majority of females (72.25%) had never heard of emergency contraception. The mean ± SD of knowledge was 14.31 ± 3.47. The overall participants' knowledge score of contraceptive methods was good in 69.8% and poor in 30.2% (Table 2).

**Table 2:** Knowledge regarding the different types of contraception (N = 400) All values are presented as numbers (N) and percentages (%) IUD, intrauterine device; UTI, urinary tract infection; SD, standard deviation

Statement		N	%
Understand the concept of family planning	No	76	19.00
	Yes	324	81.00
The well-known types of contraceptive methods	Contraceptive pills	341	85.25
	IUD	231	57.75
	Male condom	136	34.00
	Lactation	117	29.25
	Injection	104	26.00
	Patches	92	23.00
	Tubal ligation	84	21.00
	Implant method	84	21.00
	Calendar method	40	10.00
	Female condom	35	8.75
	Cervical ring	18	4.50
	Spermicidal method	16	4.00
	Diaphragm	13	3.25
	Withdrawal method	13	3.25
	Vasectomy	7	1.75
The most effective method in preventing pregnancy	Contraceptive pills	99	24.75
	Intrauterine device	53	13.25
	Male and female condoms	42	10.50
	Spermicidal	22	5.50
	Cervical ring	21	5.25
	Do not know	163	40.75
Contraceptive method that helps prevent infections	Contraceptive pills	42	10.50
	Intrauterine device	34	8.50
	Male and female condoms	119	29.75
	Spermicidal	40	12.46
	Cervical ring	33	8.25
	Do not know	132	33.00
Permanent contraceptive methods	Contraceptive pills	33	8.25
	Tubal ligation	79	19.75
	Patches	64	16.00
	Injection	25	6.25
	Vasectomy	4	1.00
	Do not know	195	48.75
Side effects of hormonal contraceptives	Mood swing	288	72.00
	Vaginal infection/UTI	235	58.75
	Menorrhagia	212	53.50
	Acne vulgaris	211	52.75
	Weight gain	209	52.25
	Unwanted pregnancies	204	51.00
	Metrorrhagia	191	47.75
	Dysmenorrhea	178	44.50
	Decrease libido	173	43.25
	Abortion	163	40.75
	Delayed recovery of fertility/infertility	143	35.75
	Amenorrhea	125	31.25
	Ectopic pregnancy	123	30.75
Background on emergency contraception	Yes	111	27.75
	No	289	72.25
Knowledge (mean ± SD)	14.31 ± 3.47		
The overall knowledge scores	Good (median of ≥15)	279	69.80
	Poor (median of <15)	121	30.20

**Figure 1:**
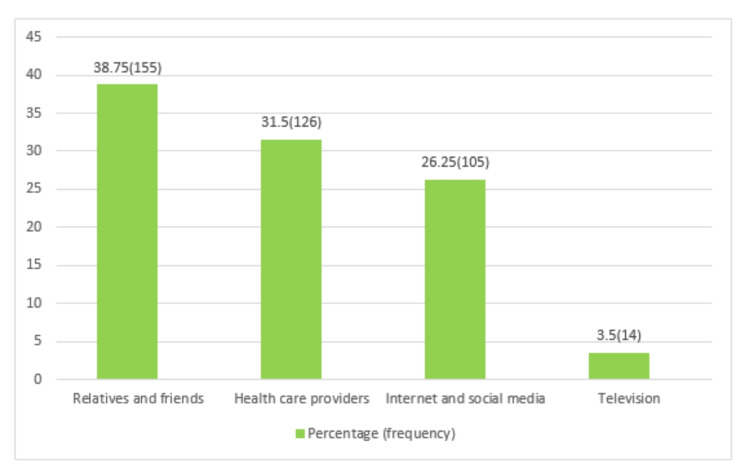
Source of information regarding contraceptive methods

Relatives and friends (38.75%), healthcare providers (31.5%), the internet and social media (26.25%), and television (3.5%) were the most popular sources of females' information about family planning (Figure 1).

Family planning has a good effect on family and societal levels, according to almost 90% and 75% of females, respectively. Half of females said that their husbands did not mind birth spacing, whereas the majority (88%) thought that husbands should be aware of and supportive of the use of contraception. About half of the study subjects (53%) reported that contraception is helpful. Despite the fact that 85.5% of females thought that family planning is important for family stability, 55.75% saw that a family with many sons is more respectable than a family with many females. The mean ± SD of females' attitude was 12.15 ± 2.62. The overall attitude score toward contraceptive methods was positive among 85% of the study sample and negative among 15% (Table 3).

**Table 3:** The attitude toward the utilization of contraceptives (N = 400) All values are presented as numbers (N) and percentages (%) SD: standard deviation

Statement		N	%
Family planning can impact on the family level	Good	359	89.75
	Bad	41	10.25
Family planning can impact on the society	Good	300	75.00
	Bad	50	12.50
	No effect	50	12.50
The husband should know about his wife's usage of contraceptives	No	56	14.00
	Yes	344	86.00
Husbands' beliefs regarding birth spacing	Disagree	114	28.50
	Do not mind	207	51.75
	Encourage	79	19.75
Females' beliefs about contraceptives	Planned to use contraceptives in the future	34	8.50
	Contraception is useful	212	53.00
	Contraception reduces sex	63	15.75
	Contraceptives are better than abortion	45	11.25
	Condoms can slip off during intercourse	46	11.50
Spouse discussion about family planning is important	Disagree	48	12.00
	Agree	352	88.00
Having an interest in knowing family planning	Disagree	74	18.50
	Agree	326	81.50
Family planning is important for females' health	Disagree	68	17.00
	Agree	332	83.00
Family planning is important for family stability	Disagree	58	14.50
	Agree	342	85.50
The large family size affects the development of the family	Disagree	79	19.75
	Agree	321	80.25
Having an interest in using family planning	Disagree	85	21.25
	Agree	315	78.75
Helping in counseling of other females for family planning	Disagree	80	20.00
	Agree	320	80.00
Having many children is a family asset	Disagree	117	29.25
	Agree	283	70.75
A family with many sons is more respectable than a family with many females	Disagree	177	44.25
	Agree	223	55.75
Usage of family planning is incorrect issue	Disagree	333	83.25
	Agree	67	16.75
Attitude (mean ± SD)	12.15 ± 2.62		
The overall attitude scores	Positive (median of ≥13)	340	85.00
	Negative (median of <13)	60	15.00

Over 80% of females have utilized any form of contraceptives to get the benefit of spacing out their pregnancies by 35.52% or completing their families by about 31.15%. Being recently married (25.32%), wanting to have more children (22.78%), and being concerned about side effects (21.52%) were the top three reasons given for not using contraceptives. Almost half of the study sample (44.24%) had been using contraceptives for two to five years. The most frequent negative effects of contraceptives among females who used them were weight gain (29.59%) and mood swings (28.04%), followed by menorrhagia (25.86%). Two-thirds of females who used family planning did so on the recommendation of their doctors (Table 4).

**Table 4:** Utilization of contraceptives among the study subjects All values are presented as numbers (N) and percentages (%) UTI: urinary tract infection

Statement		N	%
History of the utilization of any contraceptive method (N = 400)	No	79	19.75
	Yes	321	80.25
Reason for using contraceptives (N = 321)	Complete the family	100	31.15
	Spacing births	114	35.52
	Improving health	89	27.72
	Economic benefits	18	5.61
Reason for not using contraceptives (N = 79)	Want more children	18	22.78
	Fear of side effects	17	21.52
	Newly married	20	25.32
	Husband's opposition	10	14.29
	Lack of knowledge	14	20.00
The duration of contraceptive use in years (N = 321)	Two to five years	142	44.24
	More than five years	42	13.08
	A year or less	93	28.97
	Do not remember	44	13.71
The most frequent side effects of family planning methods (N = 321)	Weight gain	95	29.59
	Mood swing	90	28.04
	Menorrhagia	83	25.86
	Decrease libido	53	16.51
	Metrorrhagia	45	14.02
	Vaginal infection/UTI	33	10.28
	Acne vulgaris	26	8.10
	Delayed recovery of fertility/infertility	23	7.17
	Dysmenorrhea	19	5.92
	Amenorrhea	17	5.29
	Abortion	15	4.67
	Unwanted pregnancies	11	3.43
	Ectopic pregnancy	4	1.25
Usage of contraceptives based on a prescription from the physician (N = 321)	Yes	219	68.22
	No	102	31.77

**Figure 2:**
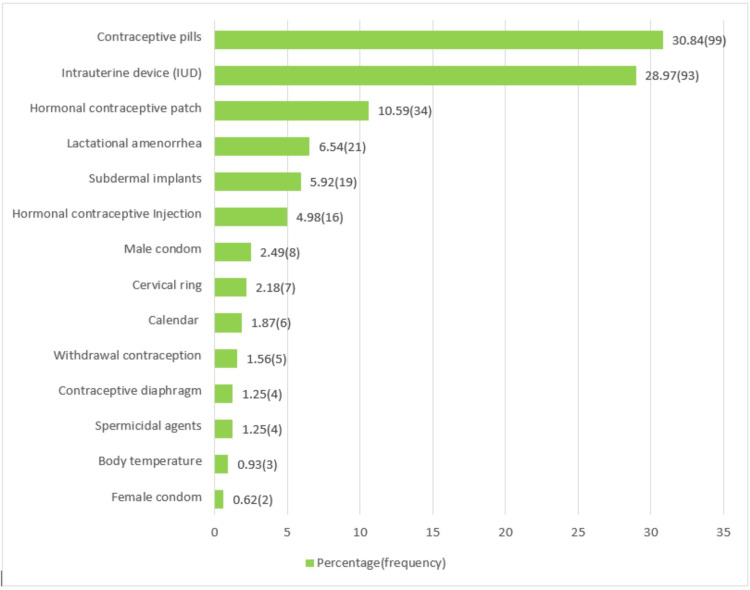
The utilized contraceptive methods among the study subjects

Contraceptive pills (30.84%), IUDs (28.97%), and patches (10.59%) were the most often used forms of contraception, with the other forms being used at rates of 6.54% and lower (Figure 2).

A binary logistic regression analysis of the relationship between demographics, obstetric experience, and knowledge of contraception found that young age and urban residence were significant predictors of the research respondents' better knowledge of contraceptive techniques (P = 0.01, OR = 0.14, 95% CI = 0.03-0.65 and P = 0.01, OR = 0.24, 95% CI = 0.09-0.68, respectively) (Table 5).

**Table 5:** The association between demographic factors, maternity experience, and contraceptive knowledge (N = 400) P ≤ 0.05 is statistically significant *Reference OR, odds ratio; CI, confidence interval; SAR, Saudi Arabian Riyal

Statement		P value	OR	95% CI for OR	
				Lower	Upper
Age group (years)	18-25	0.01	0.14	0.03	0.65
	26-30	0.15	0.36	0.09	1.43
	31-40	0.11	0.35	0.10	1.26
	41-49*				
Nationality	Saudi	0.42	1.34	0.66	2.69
	Non-Saudi*				
Residency	Ahad Bani Zayd	0.80	0.86	0.26	2.84
	Alardyat	0.25	0.51	0.16	1.59
	Al-Qunfudah City	0.01	0.24	0.09	0.68
	Al Quoz	0.71	0.82	0.28	2.38
	Haly	0.32	0.56	0.17	1.78
	Sabt Aljarah*				
Education	Reads and writes	0.15	0.27	0.05	1.58
	Elementary school	0.25	0.35	0.06	2.14
	Middle school	0.43	0.57	0.14	2.32
	High school	0.62	0.72	0.20	2.63
	University level	0.26	0.50	0.15	1.68
	Above university level*				
Employment status	Housewife	0.78	1.09	0.59	2.04
	Employee	0.21	1.74	0.73	4.17
	Student*				
Monthly income in SAR	<5,000	0.35	0.70	0.33	1.48
	5,000-10,000	0.44	0.80	0.46	1.41
	>10,000*				
The duration of marriage in years	≤1	0.80	1.14	0.43	3.02
	>1-10	0.74	0.89	0.44	1.78
	>10*				
Birth spacing in years	1	0.88	1.12	0.25	4.99
	2-3	0.96	0.97	0.27	3.48
	4-5	0.33	0.56	0.18	1.80
	>5*				
Number of pregnancies	None	0.45	0.48	0.07	3.20
	1-2	0.32	1.85	0.56	6.14
	3-4	0.93	0.95	0.32	2.82
	>4*				
Number of live children	None	0.09	3.87	0.81	18.45
	1-4	0.12	1.89	0.84	4.27
	>4*				

The relationship between demographics, obstetric history, and attitude toward contraception was explored using binary logistic regression analysis, which revealed that middle and high school educational levels were factors associated with a favorable attitude toward contraceptive methods (P = 0.02, OR = 0.17, 95% CI = 0.04-0.75 and P = 0.03, OR = 0.23, 95% CI = 0.06-0.88, respectively). Monthly income was the second important factor affecting females' attitude (P = 0.04, OR = 0.44, 95% CI = 0.20-0.96) (Table 6).

**Table 6:** The relationship between demographic, maternity background, and attitude toward contraceptives (N = 400) P ≤ 0.05 is statistically significant *Reference OR, odds ratio; CI, confidence interval; SAR, Saudi Arabian Riyal

Statement		P value	OR	95% CI for OR	
				Lower	Upper
Age group (years)	18-25	0.33	0.48	0.11	2.12
	26-30	0.35	0.53	0.14	1.99
	31-40	0.45	0.63	0.19	2.10
	41-49*				
Nationality	Saudi	0.56	1.24	0.61	2.53
	Non-Saudi*				
Residency	Ahad Bani Zayd	0.29	1.92	0.58	6.43
	Alardyat	0.88	0.91	0.28	2.96
	Al-Qunfudah City	0.51	1.42	0.50	4.04
	Al Quoz	0.87	1.09	0.37	3.19
	Haly	0.88	0.91	0.28	2.95
	Sabt Aljarah*				
Education	Reads and writes	0.38	0.44	0.07	2.74
	Elementary school	0.08	0.19	0.03	1.19
	Middle school	0.02	0.17	0.04	0.75
	High school	0.03	0.23	0.06	0.88
	University level	0.98	0.98	0.27	3.51
	Above university level*				
Employment status	Housewife	0.55	1.22	0.63	2.36
	Employee	1.00	1.00	0.41	2.42
	Student*				
Monthly income in SAR	<5,000	0.04	0.44	0.20	0.96
	5,000-10,000	0.63	1.15	0.65	2.05
	>10,000*				
The duration of marriage in years	≤1	0.05	0.37	0.14	1.02
	>1-10	0.97	0.99	0.49	2.00
	>10*				
Birth spacing in years	1	0.19	2.70	0.60	12.07
	2-3	0.77	0.83	0.23	2.96
	4-5	0.63	0.75	0.23	2.41
	>5*				
Number of pregnancies and number of live children	None	0.98	0.98	0.15	6.36
	1-2	0.19	0.45	0.14	1.47
	3-4	0.60	0.75	0.25	2.19
	>4*				
Number of live children	None	0.67	0.72	0.16	3.23
	1-4	0.48	1.35	0.59	3.12
	>4*				

## Discussion

Birth-to-pregnancy intervals of around 18 months or shorter are associated with an elevated risk of infant, neonatal, and perinatal mortality; low birth weight; small size for gestational age; and preterm delivery, according to the World Health Organization [4]. We undertook this study to evaluate the knowledge on, attitude toward, and practice of contraception methods among females in the Al-Qunfudah governorate because they are thought to be the primary drivers of family planning success. This study discovered that overall good knowledge about contraceptive methods was clearly found in 69.8% of the study group, which is like that obtained by a previous study among Muslim females in Nepal [17]. Additionally, 81% of the study population had a solid understanding of the family planning concept, and these encouraging findings are consistent with those of several studies conducted in Saudi Arabia (80.6%), Pakistan (81%), and India (82.2%) [15,18,19]. Additionally, Mubashar et al. [20] in the Aseer region of Saudi Arabia discovered that 99.2% of females were aware of contraception, and Tilahun et al. [21] in their study in Ethiopia found that the concept of family planning was well-known to respondents (94%). These studies had participants with similar levels of education, and prior research has shown that as education levels rise, the understanding of family planning also rises considerably [12].

In this study, the females cited several forms of contraception, including contraceptive pills (85.25%), IUDs (57.75%), and male condoms (34%). This result is like that of other cross-sectional surveys conducted in primary healthcare facilities in Al-Qassim and Abha, Saudi Arabia [12,15]. The key reason for this finding is that contraceptive pills are commonly available in pharmacies and easily used by females, making them the most popular and known form of contraception among females. Different contraceptive methods have an average failure rate ranging from less than 1% in the case of subdermal implants and combined injectable contraceptives to 21% in female condoms [2,22-26]. Regrettably, none of the females were able to determine the contraceptives that have the highest success rate in preventing pregnancy, which are the subdermal implant of progestin and combined injectable contraceptives [2]. This information is important for females to know in order to base their choices on the ability of the family planning method to protect against unwanted pregnancy.

Male and female condoms are contraceptive methods that can protect partners against sexually transmitted infections (STIs) [26]. Only one-third (29.75%) of the survey participants knew that barrier techniques such as male and female condoms could shield couples from the spread of STIs. Nearly half of the study subjects (48.75%) were unable to identify permanent contraceptive techniques, and only 19.75% of them mentioned tubal ligation. This outcome may be due to the limited uses of this form of contraception, in addition to the Islamic culture that accepts only a temporary delay of pregnancy and rejects permanent sterilization except in certain medical indications, making them unfamiliar to most females. Contraceptive techniques, like all drugs, may have side effects that are harmful to the health of females. Most of these side effects were listed by the study participants, who focused on mood swings (72%), followed by vaginal infection/UTI (58.75%) and menorrhagia (53.5%). This result is consistent with what Alhusain et al. discovered in their study conducted in Jeddah, Saudi Arabia, in 2018; their study sample of females mentioned the same negative impacts of the family planning methods [13]. Just 27.75% of study participants were aware of emergency contraception. This result is the same as that of another Saudi Arabian survey, which found that only 31.6% of respondents knew that there is a method to prevent pregnancy after unprotected intercourse [10]. Hence, more efforts are still required to raise females' understanding of various family planning options and their advantages for their health, as well as to dispel myths about their adverse effects.

Most of the females in this study learned about family planning methods from their parents, relatives, and friends (38.75%) and then from healthcare providers (31.5%). This finding is similar to that of other earlier Saudi studies [12,17,27,28], and this result may be due to Saudi cultural norms and a lack of a school curriculum that teaches students about reproductive health issues, including birth spacing and its benefits for females and child health. Based on this important discovery, healthcare professionals should be given more authority and instructions to inform females about family planning options. Also, reproductive health topics should be included in the educational curriculum to improve female students' awareness of them.

In our survey, 89.75% of participants acknowledged family planning's beneficial effects on individuals, families, and communities. This conclusion is corroborated by a different study, which found that family planning had a significant positive impact on population health and well-being [2]. Only 19.75% of the females in this study reported that their husbands were aware of their family planning methods, which is much lower than another study that reported that 41% of husbands voiced approval of family planning [12]. Furthermore, partner discussion about the method of family planning was recommended by 88% of participants in this study, which is similar to another Saudi study that reported that most of the females only wanted to use family planning in agreement with their spouses [15]. This conclusion is very crucial, and the spouses need to be made more aware that the choice to take contraceptives is one that they both have to make, not only the females.

The current study found that 80.25% of the participants have used any form of contraception; this estimate is roughly in line with a cross-sectional study carried out in Hail City (85%) in 2020 [29], but it is slightly higher than those in Jazan in 2018 (64.4%) [30], Taif in 2015 (67.7%) [11], and Jeddah in 2018 (69.7%) [13]. The utilization of contraception was similar in these Saudi areas due to the similarities in the participants' socio-economic characteristics. The main reason for using contraceptives in this study is child spacing (35.96%); this finding is supported by the researchers in Jazan in 2018 [30]. Contraceptive pills were the most widely used method of birth control, followed by intrauterine devices (28.97%). This pattern resembles that of a Saudi study conducted in Taif [11], but it differs from that of a study conducted in Nigeria, which found that injectable family planning methods (39.2%) and pills (32.1%) were the most widely used family planning strategies [31]. Overall, the research revealed that a low percentage of the participants (2.49%) depended on male condoms as a contraceptive method; this result is consistent with one from an Indian study [32]. Oral contraceptive pills are easily used by females, so they are the most frequently used family planning methods, while the male condom needs more attention from both partners to avoid destruction during sexual intercourse, and its use depends on the husband's motivation and agreement, which is rarely achieved.

In our study, females who used contraceptive methods most frequently experienced weight gain, mood fluctuations, and metrorrhagia as adverse effects. However, according to a descriptive cross-sectional study conducted in 2016 among Saudi females in Jeddah, Saudi Arabia, mood swings or depression was the most common side effect of contraception, occurring in 34.6% of cases, followed by weight gain in 23.3% of cases [13]. This discrepancy in both studies' findings is not surprising, as the side effects of any medication, including family planning methods, are individualized and vary from one female to another.

Younger age and urban residency of the females were shown to be factors associated with their better understanding of contraception in this study, which contrasts with a previous Saudi study that found older females having better knowledge of family planning [12]. Our explanation for this finding is that young females and those living in urban areas may have more opportunity to be highly educated, attend health education campaigns, or even have more internet access to learn more about health-related issues in comparison with older females and those living in rural areas. Instead, it was found that females with lower monthly incomes and who had middle and high school educations had significantly higher positive attitudes toward contraception. This finding is contrary to what was concluded in a previous Saudi study, which revealed that positive attitudes toward family planning were higher among females with a higher socio-economic status [33]. This disparity might be explained by the different study techniques and sample selection.

This study has some limitations that should be taken into consideration. First, only educated females who are accustomed to utilizing internet technologies and have internet connections would be able to complete the online, self-administered questionnaire; thus, the study ignored the illiterate females and those who did not have internet access. Second, the cross-sectional design provides a constrained, cursory view of the females' perspectives on family planning. Third, the use of a convenient sample may limit the generalization of the study results, and we recommend doing further studies on this issue using random sampling techniques. Despite the restrictions already indicated, this study is an initiative for more qualitative research in this distant area.

## Conclusions

According to the study, females have generally satisfactory knowledge of and a positive attitude toward various forms of contraception. Oral contraceptive tablets and IUDs were the two contraception methods that were most often used. Weight gain, mood swings, and menorrhagia were the most frequently encountered adverse effects of family planning methods. Young age and urban residency were associated with good knowledge about contraception, while low income and holding middle and high school educational degrees were factors for a positive attitude toward contraception use. As a result, we recommend making consistent efforts to apply health education sessions for both females and males to increase their awareness toward different contraceptive modalities, with a focus on emergency and permanent contraception, and dispel myths about their adverse effects. This may be achieved by enhancing the doctor-patient relationship, increasing public awareness through campaigns, offering information sources, and streamlining the process of acquiring such resources. We think it is critical for religious experts to take a proactive role in educating the general population about the religion's perspective on the subject of family planning in order to eliminate misconceptions among couples regarding the Islamic religion's restriction on using certain contraceptive options. Family planning clinics should be more available in the governmental primary healthcare setting to be accessible to all females and to ensure professional contraception counseling for them. Finally, further qualitative research in the form of focus group discussions or in-depth interviews with both females and their husbands will allow a deep understanding of both partners' perspectives on the different types of family planning methods.
